# An Efficient Strategy for Obtaining Mutants by Targeted Gene Deletion in *Ophiostoma novo-ulmi*

**DOI:** 10.3389/fmicb.2021.699783

**Published:** 2021-07-14

**Authors:** Jorge Luis Sarmiento-Villamil, Thais Campos de Oliveira, Erika Sayuri Naruzawa, Louis Bernier

**Affiliations:** ^1^Centre d’Étude de la Forêt (CEF) and Institut de Biologie Intégrative et des Systèmes (IBIS), Université Laval, Quebec City, QC, Canada; ^2^Collège Communautaire du Nouveau-Brunswick, Réseau CCNB-INNOV, Grand Falls, NB, Canada

**Keywords:** gene deletion method, Dutch elm disease, non-homologous end joining, counterselection, *Ophiostoma novo-ulmi*

## Abstract

The dimorphic fungus *Ophiostoma novo-ulmi* is the highly aggressive pathogen responsible for the current, highly destructive, pandemic of Dutch elm disease (DED). Genome and transcriptome analyses of this pathogen previously revealed that a large set of genes expressed during dimorphic transition were also potentially related to plant infection processes, which seem to be regulated by molecular mechanisms different from those described in other dimorphic pathogens. Then, *O. novo-ulmi* can be used as a representative species to study the lifestyle of dimorphic pathogenic fungi that are not shared by the “model species” *Candida albicans* and *Ustilago maydis.* In order to gain better knowledge of molecular aspects underlying infection process and symptom induction by dimorphic fungi that cause vascular wilt disease, we developed a high-throughput gene deletion protocol for *O. novo-ulmi*. The protocol is based on transforming a Δ*mus52 O. novo-ulmi* mutant impaired for non-homologous end joining (NHEJ) as the recipient strain, and transforming this strain with the latest version of OSCAR plasmids. The latter are used for generating deletion constructs containing the toxin-coding *Herpes simplex* virus thymidine kinase (*HSVtk*) gene which prevents ectopic integration of the T-DNA in *Ophiostoma* DNA. The frequency of gene deletion by homologous recombination (HR) at the *ade1* locus associated with purine nucleotide biosynthesis was up to 77.8% in the Δ*mus52* mutant compared to 2% in the wild-type (WT). To validate the high efficiency of our deletion gene methodology we deleted *ade7*, which also belongs to the purine nucleotide pathway, as well as *bct2*, *ogf1*, and *opf2* which encode fungal binuclear transcription factors (TFs). The frequency of gene replacement by HR for these genes reached up to 94%. We expect that our methodology combining the use of NHEJ deficient strains and OSCAR plasmids will function with similar high efficiencies for other *O. novo-ulmi* genes and other filamentous fungi.

## Introduction

Native elm populations from Europe and North America were devastated by two pandemics of Dutch elm disease (DED), a vascular wilt disease caused by invasive Ascomycete fungi in the genus *Ophiostoma* (Brasier, 1979, 1991; Santini et al., 2012). The current DED pandemic, which has been going on since the 1940s, is caused by the highly aggressive *Ophiostoma novo-ulmi* (Brasier, 1991). Since the DED pandemics, European elms have lost their roles of dominant riparian forest trees and are used only scarcely in forest restoration or urban planting. However, the field elm (*Ulmus minor*) survived in many locations because of its considerable ability to sprout and produce root suckers, even though young trees show recurrent DED symptoms once they reach 3–5 m in height (Martín et al., 2019). A similar situation was reported for American elms in North America (Parker and Leopold, 1983). This situation, coupled with loss of elms due to human activities, indicates a high risk of extinction in several elm populations. DED pathogens are transmitted from diseased to healthy elms by elm bark beetles from the *Scolytus* and *Hylurgopinus* genera (Parker et al., 1948; Santini and Faccoli, 2015). According to the current model of infection by these dimorphic organisms, the fungus colonizes xylem elements by both budding and hyphal growth: the unicellular yeast form facilitates passive vertical spread within xylem vessels of elm trees, whereas the multicellular mycelium penetrates bordered pits and invades uninfected adjacent vessels. Mycelium is also associated with saprophytic growth of DED fungi in breeding galleries excavated by bark beetles under the bark of moribund or recently killed trees. During pathogenesis, DED fungi can produce cell wall degrading enzymes (CWDEs) that facilitate the invasion of tissues, and the release of sugars that can be used as nutrients. The tree reacts to infection by a massive blocking of vessels with tyloses that, together with fungal dimorphic growth and secretion of effectors and secondary metabolites such as toxins, induces embolisms in functional vessels. These processes lead to wilting and necrosis of plant tissues (Bernier, 2016).

In spite of the economic and ecological impacts of DED pathogens, few studies have provided insight into the molecular and cellular mechanisms that control vegetative and parasite growth of these species. However, the sequencing and annotation of a reference genome for *O. ulmi* and *O. novo-ulmi* identified putative orthologs of genes involved in dimorphic growth, secondary metabolite biosynthesis, effector repertoires and other pathogenicity and/or virulence factors (Forgetta et al., 2013; Khoshraftar et al., 2013; Comeau et al., 2015). These genomic analyses have provided a valuable means for predicting cellular processes associated with DED development. In pathogenic fungi, control of cell differentiation and secondary metabolism is often coordinated simultaneously by the same molecular mechanisms, and dimorphic transition is required to complete infection and induce disease symptoms (Nadal et al., 2008; Boyce and Andrianopoulos, 2015). Interestingly, transcriptomic analysis of yeast to hypha transition in *O. novo-ulmi* provided evidence that a large set of homologous genes associated with pathogenicity and virulence were differentially expressed during this morphological change (Nigg and Bernier, 2016). For example, 15 key homologous genes encoding proteins linked to mitogen activated protein kinase pathways were induced under this condition. Theses pathways play important roles in morphogenetic development and infection process (Nadal et al., 2008; Turrà et al., 2014). It should also be underlined that gene OphioH237g7309, encoding a fungal binuclear transcription factor (TF) associated with cluster regulation, exhibited the highest level of induction among genes encoding this type of regulatory proteins during multicellular mycelium growth of *O. novo-ulmi.* This TF belongs to a fujikurin-like gene cluster (OpPKS8) that is unique to DED pathogens within the Ophiostomatales, but whose presence is widespread among phytopathogens and plant-associated fungi (Sbaraini et al., 2017). Comparative analysis of transcriptomes of *O. novo-ulmi* and model dimorphic species *Candida albicans* and *Histoplasma capsulatum* showed few similarities in gene expression patterns between yeast and mycelial growth (Nigg et al., 2015). Taken together, these works suggest that dimorphic growth could be closely related to DED infection development, but regulated by other molecular mechanisms in *Ophiostoma* species, which offers a new perspective for understanding the relationship between pathogenesis, secondary metabolism and dimorphic growth.

The identification of large sets of genes whose roles are worthy of functional exploration requires a highly efficient system for gene deletion. However, the efficiency of gene knockout protocols developed so far for *O. novo-ulmi* is remarkably low and needs to be substantially improved in order to fully exploit new knowledge and validate novel hypotheses proposed from next generation sequencing (NGS) approaches (Bernier, 2016). Genes CU, *epg1*, and *mus52* were inactivated by targeted disruption in less than 2% of the transformants obtained from *O. novo-ulmi* (Bowden et al., 1996; Temple et al., 2009; Naruzawa, 2015). This low efficiency of gene disruption by homologous recombination (HR) is commonly encountered in filamentous fungi, because non-homologous end joining (NHEJ) is the primary mechanism for exogenous DNA integration in the fungal genome, despite the presence of long stretches of homologous sequence between transforming and genomic DNA (Chang et al., 2017). Among key proteins involved in NHEJ, Ku70 and Ku80 form a heterodimer that binds to exogenous DNA, thereby acting as a platform for the subsequent recruitment of protein complexes, such as the XRCC4-DNA ligase IV complex, which is then targeted to the break in order to integrate the exogenous DNA into the host genome and seal the gap (Dudásová et al., 2004; Gelvin, 2017). Generally, the rate of homologous integration has been improved by blocking the NHEJ pathway in a broad range of fungi, including important pathogens (Kooistra et al., 2004; Ninomiya et al., 2004; Nayak et al., 2006; Choi and Shim, 2008; Choquer et al., 2008; Snoek et al., 2009; Xu et al., 2014). Furthermore, the deletion of *Ku* genes in these organisms does not affect their vegetative growth and ability to cause disease (Ninomiya et al., 2004; Choi and Shim, 2008; Haarmann et al., 2008; Li et al., 2010; Xu et al., 2014). Deletion of either *mus51* or *mus52*, which code for the Ku70 and Ku80 proteins, increased the frequency of accurate gene targeting up to 100% in *Neurospora crassa*, *Kluyveromyces lactis*, *Aspergillus nidulans*, and *Cryptococcus neoformans* (Kooistra et al., 2004; Ninomiya et al., 2004; Nayak et al., 2006). In strains of *Fusarium verticillioides, Claviceps purpurea*, and *Penicillium chrysogenum* that lacked Ku70 activity, HR rates of 30–60, 30–50, and 56% were obtained, respectively (Choi and Shim, 2008; Haarmann et al., 2008; Snoek et al., 2009). Naruzawa (2015) reported that a Δ*mus52* mutant of *O. novo-ulmi* did not show any alteration in vegetative development and pathogenicity, while showing increased gene replacement by HR at the *ppo1* locus encoding a putative cyclooxygenase.

In spite of enhancement of HR events in NHEJ-defective strains, extensive screening is still required to ensure replacement at the desired locus. This step is overall quite laborious and time-consuming, and thus limits systematic gene disruption and genomic functional analysis. A high efficiency deletion gene protocol based on HR also requires a high-throughput approach to generate deletion constructs and simplify the identification of deletion mutants upon fungal transformation.

Generally, production of a gene deletion construct requires various cloning steps to assemble the HR cassette, followed by its insertion into a backbone vector (García-Pedrajas et al., 2013). OSCAR (One Step Construction of *Agrobacterium* Recombination ready plasmids) was developed as a very simple method to produce gene deletion constructs for fungi compatible with *Agrobacterium tumefaciens*-mediated transformation (ATMT) in one cloning step in 2 days (Paz et al., 2011). This method involves PCR amplification of the upstream and downstream sequences flanking the gene of interest, using gene specific primers each containing one of four different attB recombination sites at its 5′ end. Furthermore, the MultiSite Gateway^®^ cloning system has been adapted so that a single cloning step of these PCR-amplified gene flanks with specifically designed marker and binary vectors generates the final deletion construct (Paz et al., 2011; García-Pedrajas et al., 2013). The latest OSCAR update carries the negative selection marker of *Herpes simplex* virus thymidine kinase (*HSVtk*) to prevent ectopic integration of the T-DNA harboring the deletion construct by supplementing the nucleoside analog 5-fluoro-2′-deoxyuridine (5FU) to the culture medium (Sarmiento-Villamil et al., 2020). Ectopic transformants expressing *HSVtk* are able to convert 5FU to 5-Fluoro-2′-deoxyuridine-5-monophosphate (5FUMP) that, when incorporated into DNA, blocks activity of thymidylate synthase, resulting in inhibition of 2′-deoxythymidine-5-triphosphate (dTTP) synthesis and leading to loss of the dTTP pool and fungal cell death (Amelina et al., 2016). This OSCAR version was validated with the deletion of genes *VdRGS1, vrg1*, and *vvs1* in *Verticillium dahliae.* PCR analyses were performed on 30 transformants, and all but one showed deletion of the target gene (Sarmiento-Villamil et al., 2020). *HSVtk* was also confirmed to work successfully in counterselection of ectopic transformants and enriching for gene deletion mutants in several ascomycetes including *Magnaporthe grisea*, *Fusarium oxysporum*, and *Fusarium graminearum* (Gardiner and Howlett, 2004; Khang et al., 2005; Twaruschek et al., 2018).

In the work described herein, we used the OSCAR-based counterselection system in combination with a Δ*mus52* mutant defective in NHEJ to develop an efficient strategy for systematic gene deletion in the highly aggressive DED fungus *O. novo-ulmi*. Although these two methods can be used separately, the NHEJ mutant exhibited a significant increase in the frequency of HR [over 75% compared to 2% in wild-type (WT)] when using the counterselection marker approach. We first targeted genes *ade1* and *ade7* since their deletion resulted in the production of characteristic pink mutants on non-selective (rich) media, thereby facilitating the phenotypic identification of knockout mutants. Then, we validated our strategy by successfully deleting genes *bct2*, *ogf1*, and *opf2* which encode TFs belonging to the Zn(II)2Cys6 family. These new efficient tools for targeted gene deletion, together with existing resources for the study and manipulation of DED fungi, open the way to a new level of functional analysis and understanding the relationship between morphogenesis and pathogenesis in vascular tree pathogens.

## Materials and Methods

### Strains, Media, and Growth Conditions

Wild-type strain *Ophiostoma novo-ulmi* subsp. *novo-ulmi* H327 (Et-Touil et al., 1999; Plourde et al., 2008) and NHEJ-defective mutant strain 174_68Δ*mus52* (Δ*mus52*) derived from it (Naruzawa, 2015) were used as parental strains to generate deletion mutants. Strains were maintained on potato dextrose agar (PDA) (Difco, #DF0013-17-6) supplemented to a final concentration of 2% agar (2PDA). Yeast cells for each parental strain were obtained by incubation for 7 days at 25°C and 250 rpm in 50 mL of liquid *Ophiostoma* minimal medium (OMM) containing L-proline as the nitrogen source (Bernier and Hubbes, 1990). Then, yeast cells of each parental strain were harvested by filtration through one layer of Miracloth (Calbiochem, #475855) and centrifugation at 5500 g for 10 min. Yeast cells were then suspended in sterile distilled water to a final concentration of 5 × 10^7^ cells/mL. Transformants expressing either of the hygromycin B phosphotransferase (*hph*) or nourseothricin acetyltransferase (*nat1*) resistance genes were cultivated on 2PDA supplemented with 300 μg/L hygromycin B (hygromycin, Thermo Fisher Scientific, #10687010) or 50 μg/L nourseothricin dihydrogen sulfate (nourseothricin, Research Products International, # N5200-0.1) as selection agents, respectively. To counterselect transformants expressing *HSVtk*, 50 μg/L 5FU (Thermo Fisher Scientific, #L16497) was added to selection medium.

The *ccd*B Survival^TM^ 2 T1^*R*^ *Escherichia coli* strain (Thermo Fisher Scientific, #A10460) was used to propagate the vector pOSCAR-HSVtk (Sarmiento-Villamil et al., 2020), which harbors the *ccdB* gene lethal for most *E. coli* strains. *E. coli* One shot^®^ OmniMAX^TM^ 2 T1^*R*^ competent cells (Thermo Fisher Scientific, #C854003) were used to produce the deletion construct. For other manipulations *E. coli* strain DH5α (Bethesda Research Laboratories, Gaithersburg, MD) was used. *E. coli* strains were grown in or on Luria Bertani (LB) media containing 100 μg/mL ampicillin sodium salt (ampicillin, Sigma-Aldrich, #A9518) or 100 μg/mL spectinomycin dihydrochloride pentahydrate (spectinomycin, Sigma-Aldrich, #S9007). Fungal parental strains were transformed with *A. tumefaciens* which was grown on LB or in minimal medium (MM) supplemented with 50 μg/mL spectinomycin. MM contained 11.5 mM K_2_HPO_4_ pH7.0, 11 mM KH_2_PO_4_ pH 7.0, 5.1 mM NaCl, 2.5 mM MgSO_4_⋅7H_2_O, 0.7 mM CaCl_2_, 9 μM FeSO_4_⋅7H_2_O, 4.4 mM (NH_4_)_2_NO_4_, 10 mM glucose, 1.7 μM ZnSO_4_⋅7H_2_O, 2.0 μM, CuSO_4_⋅5H_2_O, 8 μM H_3_BO_3_, 3.3 μM MnSO_4_ ⋅H_2_O, and 8.3 μM Na_2_MoO_4_ ⋅2H_2_O.

### Generation of Deletion Constructs

To determine the effects of the homologous arm length on the frequency of HR in WT *O. novo-ulmi* subsp. *novo-ulmi*, deletion constructs with different lengths of homologous sequence for *ade1* (OphioH327g7434) were generated using the OSCAR method but replacing the OSCAR vector with pOSCAR-HSVtk, which contains the *HSVtk* gene that prevents ectopic integration of the T-DNA (Sarmiento-Villamil et al., 2020). Constructs pade1_hyg_1kb and pade1_hyg_2kb were obtained with 1 Kb and 2 Kb of DNA homologous to each region flanking *ade1*, respectively. Each region flanking *ade1* was amplified using the corresponding primer pairs listed in Supplementary Table 1 and Platinum *Taq* polymerase high fidelity (Thermo Fisher Scientific, #11304011). PCRs were performed according to the manufacturer’s recommendations in a total volume of 50 μL and under the following PCR cycling conditions: an initial denaturation of 1 min at 94°C, 35 cycles of 30 s at 94°C, 30 s at 58°C, and 1 min/kb at 68°C, and completed with a final extension of 5 min at 68°C. All PCR products were gel-purified using EZ-10 spin column DNA gel extraction miniprep kit (Bio Basic, #BS367) according to the manufacturer’s instructions. For each deletion construct, a 5 μL BP clonase reaction was set up to include 1 μL BP clonase II enzyme mix (Thermo Fisher Scientific, #11789020), 10 ng/kb of each purified PCR flank, 95 ng of pOSCAR-HSVtk and 100 ng of pA-Hyg-GFP-OSCAR or 60 ng of marker vector pA-NTC-OSCAR. The BP reaction was incubated at 25°C for 20 h in a PCR thermocycler. Then, the reaction mixture was used to transform One Shot OmniMAX^TM^ 2 T1R Chemically Competent *E. coli* according to the manufacturer’s recommendations. Bacterial colonies were recovered on LB plates supplemented with 100 μg/mL spectinomycin following overnight incubation at 37°C. Correct structure of each deletion construct was verified by restriction enzyme digestion. Constructs pade1_hyg_1kb and pade1_hyg_2kb were developed using pA-Hyg-GFP-OSCAR.

To assess if inactivating the NHEJ pathway increases HR frequency in *O. novo-ulmi*, deletion constructs with 1 Kb or 2 Kb flanking regions for *ade1* and *ade7* (OphioH327g6779) were generated to transform *O. novo-ulmi* strain 174_68Δ*mus52.* These deletion constructs were obtained using the OSCAR method described above, but replacing the pA-Hyg-GFP-OSCAR by pA-NTC-OSCAR. Each region flanking *ade7* was amplified using the corresponding primer pairs listed in Supplementary Table 1. To validate the efficiency of the *O. novo-ulmi* strain 174_68Δ*mus52* and HSVtk-counterselection-based protocol, deletion OSCAR constructs with 1 Kb of homologous arm length for *bct2* (OphioH327g2340), *ogf1* (OphioH327g1537), and o*pf2* (OphioH327g1642) were generated using the corresponding primer pairs listed in Supplementary Table 1.

### *Agrobacterium tumefaciens*-Mediated Transformation of *Ophiostoma novo-ulmi*

*Agrobacterium tumefaciens* strain GV3101:pMP90 (Koncz and Schell, 1986) was used for transferring T-DNA harboring dual molecular selection markers. The deletion constructs were transformed into *A. tumefaciens* strain GV3101:pMP90 using the heat shock method, spread onto LB plates supplemented with 100 μg/mL spectinomycin and incubated at 25°C for 2 days.

For *A. tumefaciens*-Mediated Transformation (ATMT) of *O. novo-ulmi*, the method described by Mullins et al. (2001) was used with some variations. *A. tumefaciens* strains containing a deletion construct were grown at 28°C for 2 days in MM supplemented with 100 μg/mL spectinomycin. *A. tumefaciens* cultures were diluted to optical density (OD_600_) of 0.2 in 6 mL induction medium (IM), which contained MM supplemented with 0.5% (W/V) glycerol, 40 mM 2-(N-morpholino) ethanesulfonic acid (MES, Sigma-Aldrich, #M5287) at pH 5.3 and 100 μg/mL spectinomycin. Bacterial cells were grown for an additional length of time until the OD_600_ value reached 0.6–0.9. Then, 100 μL of each *A. tumefaciens* culture and 100 μL of a yeast cell suspension (5 × 10^8^ cells/mL) from each fungal parental strain were mixed and spread on a sterile 0.45 μm pore nitrocellulose membrane (Sartorius Stedim Biotech GmbH, #1140647ACN) overlaid on top of 20 mL of co-culture medium plate, which contained IM plus 200 μM 3′,5′-dimethoxy-3′hydroxyacetophenone acetosyringone (AS, Sigma-Aldrich, #D134406) and 2% (W/V) agar. Following incubation at 25°C for 2 days, the filters were cut in strips and transferred to selection medium, which contained 2PDA supplemented with 300 μg/mL hygromycin or 50 μg/L nourseothricin to select the transformants, with or without 50 μg/mL 5FU as a counterselecting agent of ectopic transformants and 100 μg/mL cefotaxime sodium salt (cefotaxime, Sigma-Aldrich, #C7039) and 100 μg/L moxalactam sodium salt (Sigma-Aldrich, #M5158) to kill the *A. tumefaciens* cells. After 2 days of incubation at 25°C, single colonies began to appear on the selection medium, which were transferred to 2PDA. Three independent ATMTs of each parental fungal strain were performed with each combination of *A. tumefaciens* deletion construct generated.

Phenotype analysis based on the recovery of pink colonies from individual transformants was used for distinguishing visually Ade^–^ from Ade^+^ survivors. To confirm gene replacement, randomly selected pink colonies were analyzed by PCR with primer combinations ade1F_809 and ade1R_1221, and ade7F_368 and ade7R_809 (Supplementary Table 1), which amplified roughly 441 and 594 bp of *ade1* and *ade7*, respectively.

### Pathogenicity Tests

Three-year-old saplings of American elm (*Ulmus americana*) grown from a local seed source were inoculated with *O. novo-ulmi* in a greenhouse compartment kept at 18°C during the night and 24°C during the day, with a 16-h photoperiod and 60% humidity (Et-Touil et al., 2005). Fungal treatments included WT strain H327 and three insertional mutants derived from it: 174_68Δ*mus52* (Δ*mus52*), ade1*-*6 (Δ*mus52*Δ*ade1*) and ade7-13 (Δ*mus52*Δ*ade7*). Non-inoculated control saplings were injected with sterile distilled water. Saplings were inoculated in early July 2020, after the leaves had fully expanded. Inoculations consisted in injecting 25 μl of a yeast cell suspension (2 × 10^6^ cells/mL) into a hole drilled with a 3/32 bit (Beier et al., 2017) at ca 20 cm up the main stem, with 6 to 10 biological repetitions per treatment. Inoculation holes were covered with Parafilm (Bemis Co., Inc., Neenah, WI, United States) to prevent desiccation. Saplings were watered daily for 3 weeks following inoculation. Percent defoliation was then recorded for each sapling and the mean defoliation was calculated for each treatment (Et-Touil et al., 2005).

### Statistical Analyses

The HR frequencies for each gene in each strain, treatment and replicate experiment were estimated by the pink/white transformant colony ratio and analyzed by the application of generalized linear models (GzLMs) using Logit as the link function and Binomial as the underlying distribution. An additional analysis to obtain orthogonal contrasts was also conducted among treatments with Bonferroni adjustments to the p-values. Percent disease severity on *U. americana* saplings was analyzed by application of GzLMs using identity as the link function and Gaussian as the underlying distribution. Orthogonal contrasts among the treatment medians with Bonferroni adjustments to the p-values were also performed. All statistical analyses were conducted using Lme4, optimx, Multicomp, and emmeans packages under R environment (R Project software, v. 3.6.2).

## Results

### Homologous Recombination Frequency in *Ophiostoma novo-ulmi*

**FIGURE 1:**
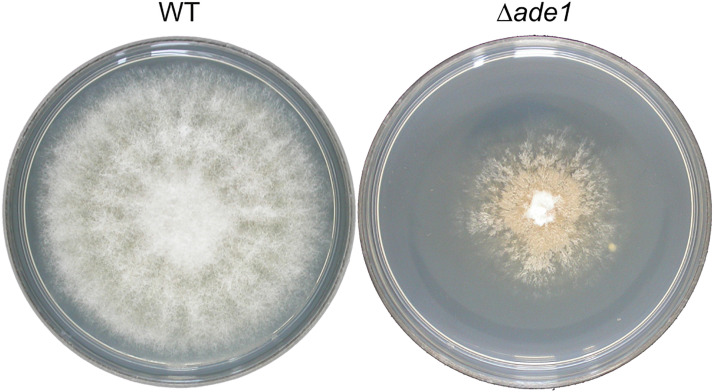
Effect of *ade1* deletion on colony morphology of *Ophiostoma novo-ulmi* after 7 days incubation on potato dextrose agar (PDA) at 21°C. The plate on the left is the parental strain, H327 (WT), and the plate on the right is a Δ*ade1* mutant.

In many filamentous fungi, production of gene-deleted strains is often limited by inefficiency of HR in these organisms. However, higher recombination efficiencies have been reported after increasing the length of homologous flanking DNA (Michielse et al., 2005). This strategy was tested in *O. novo-ulmi* by transforming WT strain H327 with *A. tumefaciens* harboring a binary vector containing a deletion construct with counterselection marker *HSVtk* located between the right border of the T-DNA and the BP clonase recombination sequence attP3, and 1 Kb or 2 Kb of region homologous to *ade1* separated by *hygR* selection marker, pade1_hyg_1kb or pade1_hyg_2kb, respectively. Gene *ade1* in *O. novo-ulmi*, located on chromosome 7, encodes a phosphoribosylamidoimidazole carboxylase (AIR carboxylase) and is a putative homolog of *ade2* from *Saccharomyces cerevisiae*, which is required for purine nucleotide biosynthesis (Myasnikov et al., 1991). As in many fungi, inactivation of *ade1* in *O. novo-ulmi* leads to auxotrophy and red pigmentation of colonies grown on non-selective (rich) media over time (Figure 1, right panel; Bernier and Hubbes, 1990). In contrast, WT strain is white-colored (Figure 1, left panel). This distinctive colony phenotype is useful as a visual signal for differentiating *ade1*^–^ from *ade1*^+^ strains when the gene is used as a marker of HR. At least 60 independent transformants per transformation experiment were randomly isolated from 2PDA supplemented with hygromycin and 5FU, which is converted to a toxic product by *HSVtk* expression (Khang et al., 2005; Wang et al., 2016). Only one out of 198 transformants obtained using pade1_hyg_2kb produced colonies that became pink over time, indicating deletion of *ade1* by HR. Transformants obtained with pade1_hyg_1kb did not produce pink colonies. PCR analysis of 18 transformants, using the primer pair ade1F-809 and ade1R-1221, confirmed the replacement of the *ade1* ORF with the *hygR* marker by absence of an amplicon only for the pink colony (data not shown). These results supported the very low HR frequency previously reported in *O. novo-ulmi* (Bowden et al., 1996; Temple et al., 2009; Naruzawa, 2015). Due to inefficiency of *HVStk* as counterselectable marker in *O. novo-ulmi*, 10 transformants that grew on 2PDA supplemented with hygromycin B and 5FU were subjected to multiplex PCR using the following primers: pGKO2_F_656, pGKO2_R_1733, pGKO2_F_2258, and pGKO2_R_2576 (Supplementary Table 1). Each PCR resulted in three fragments of roughly 1.9 Kb of the entire HVStk cassette, 1.1 Kb of the HVStk promoter proximal to the left border of the T-DNA, and 0.3 Kb of the HVStk terminator (data not shown). Eight transformants had truncations of their *HVStk* cassettes while two transformants had intact *HSVtk* cassettes (data not shown).

### Frequency of HR in a NHEJ-Impaired Mutant of *Ophiostoma novo-ulmi*

**FIGURE 2:**
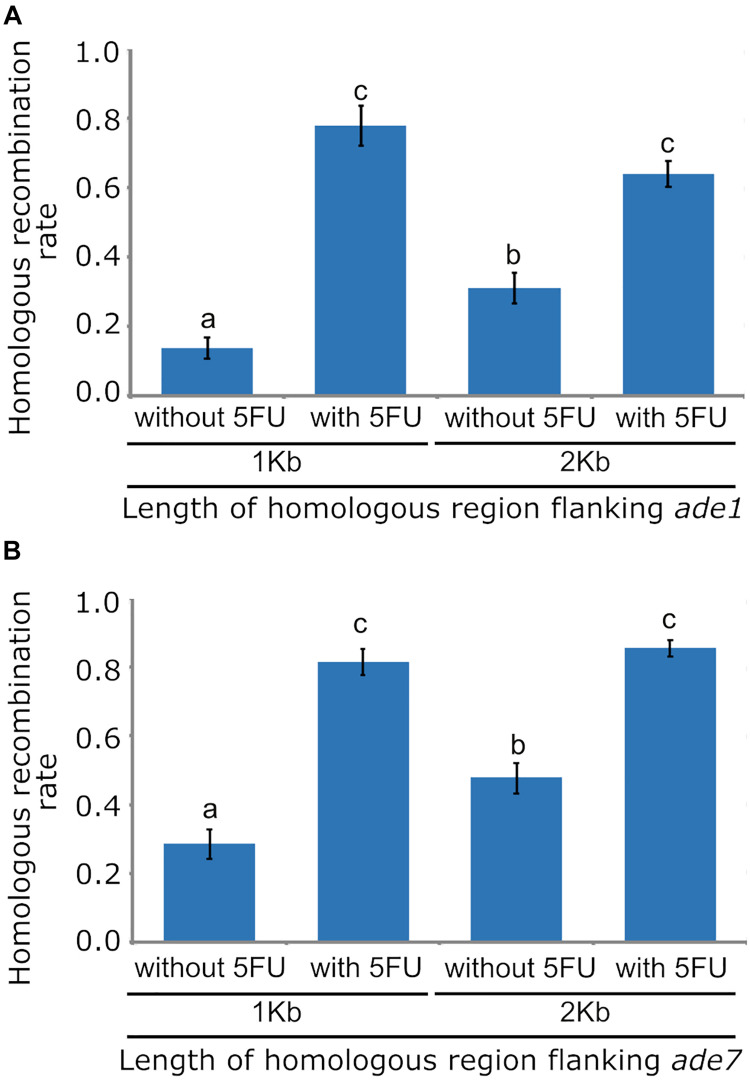
Bar graphs showing the effects of using 1 Kb or 2 Kb of homologous sequence flanking targeted loci, and applying counterselection with 5-Fluoro-2′-deoxyuridine, on the frequency of homologous recombination (HR) in *Ophiostoma novo-ulmi* strain 174_68Δ*mus52*. **(A)** HR frequency for targeted locus *ade1*. **(B)** HR frequency for targeted locus *ade7*. Standard error bars are displayed at the top of each bar. Different letters above each bar indicate statistically significant differences (*p* < 0.05) according to the sequential Bonferroni method for error correction.

Low HR frequency in fungal species has also been improved by disrupting the NHEJ DNA repair pathway, which is typically achieved by inactivating one of the principal components of this pathway, such as homologs of the Mus52/Uk80 proteins. To determine if deleting *mus52* and varying the length of homologous region increased the frequency of HR in *O. novo-ulmi*, strain 174_68Δ*mus52* was transformed with *A. tumefaciens* using pade1_NTC_1kb and pade1_NTC_2kb, which contained the *nat1* cassette inserted between 1 Kb or 2 Kb flanking regions for *ade1*, respectively. Three independent transformations of each deletion construct, using 174_68Δ*mus52* as a parental strain, produced an average of 40 transformants per transformation that were regenerated on 2PDA supplemented with nourseothricin. Phenotype analysis relying on pink colony formation in these *Mus52*-deleted transformants showed a marked increase of HR frequency in *O. novo-ulmi*. In transformants obtained with 1 and 2 Kb of flanking regions, HR frequency rose to 10.7 and 30.1%, respectively (Figure 2A). However, HR frequency was significantly higher in transformants obtained with construction containing 2 Kb of flanking homologous region, thereby showing a correlation between the length of homologous region and the efficiency of homologous integration.

### Efficiency of HSVtk-Counterselection in an NHEJ-Impaired Mutant of *Ophiostoma novo-ulmi*

Efficient selection of transformants with correct gene replacement by HR is critical for large-scale gene deletions in fungi. However, gene deletion methods generally yield mostly ectopic transformants via random T-DNA integration. Counterselection systems such as *HSVtk* and 5FU have been described for preventing random T-DNA integration in fungi. To determine the functionality of *HSVtk* counterselection in an *O. novo-ulmi* mutant impaired for NHEJ, cultures of strain 174_68Δ*mus52* were incubated with *A. tumefaciens* and plated onto 2PDA plates with nourseothricin and 5FU. An average of 30 transformants per transformation were obtained in the presence of 5FU, with an increase in HR frequency up to 63.8 and 77.8%, using deletion constructs with 1 Kb or 2 Kb homologous flanking regions, respectively (Figure 2A). There was no statistically significant difference between both HR rates. However, comparison of HR rates between transformants isolated from supplemented (5FU) versus non-supplemented medium revealed a statistically significant difference caused by a three-fold increase in the number of gene replacement mutants, as well as a stark reduction (94%) in the number of ectopic transformants in the presence of 5FU. This result showed that *HSVtk* worked well in preventing random integration of the deletion construct T-DNA in the NHEJ-impaired mutant of *O. novo-ulmi*, thus greatly simplifying the selection of null mutants.

### Confirmation of Gene Targeting Frequency in an NHEJ-Impaired Mutant of *Ophiostoma novo-ulmi*

In *O. novo-ulmi*, gene *ade7* gene located on chromosome 6 encodes a phosphoribosylaminoimidazole-succinocarboxamide synthase, which is also essential for biosynthesis of purine nucleotide, and is a putative homolog of *ade1* from *S. cerevisiae*. Mutation in *ade7* usually leads to polymerization of 5-aminoimidazole ribotide (AIR), resulting in red pigmentation of fungal cells. To confirm the effects of flanking region length and counterselection system on HR frequency of an *O. novo-ulmi* strain lacking full NHEJ activity, *ade7* was deleted in strain 174_68Δ*mus52* using deletion constructs which harbored a *nat1* cassette between 1 Kb or 2 Kb flanking regions of this gene. Cultures of strain 174_68Δ*mus52* incubated with *A. tumefaciens* were plated onto selection medium, which contained nourseothricin with or without 5FU. HR rates for transformants obtained with 1 Kb and 2 Kb flanking regions of *ade7* and isolated from selection medium without 5FU, were 28.6 and 47.7%, respectively. In comparison, HR rates in transformants isolated from selection medium supplemented with 5FU were higher (up to 80%) (Figure 2B). As expected, on selection medium lacking 5FU, the HR rate was significantly higher in transformants whose deletion construct contained a greater length of flanking homologous DNA. In addition, HR increased significantly in transformants isolated from selection medium supplemented with 5FU, showing similar trends in HR frequency as determined for *ade1* in the same conditions tested, but with a slight increase in the magnitude of HR rates (Figures 2A,B). These slight variations in HR frequencies between *ade1* and *ade7* may be closely related to locus-specific properties, including differences in chromatin structure. Recovery data for *ade7* mutants also corroborated the effect of 5FU in enrichment of gene replacement mutants as well as in decreasing the number of ectopic transformants.

**FIGURE 3:**
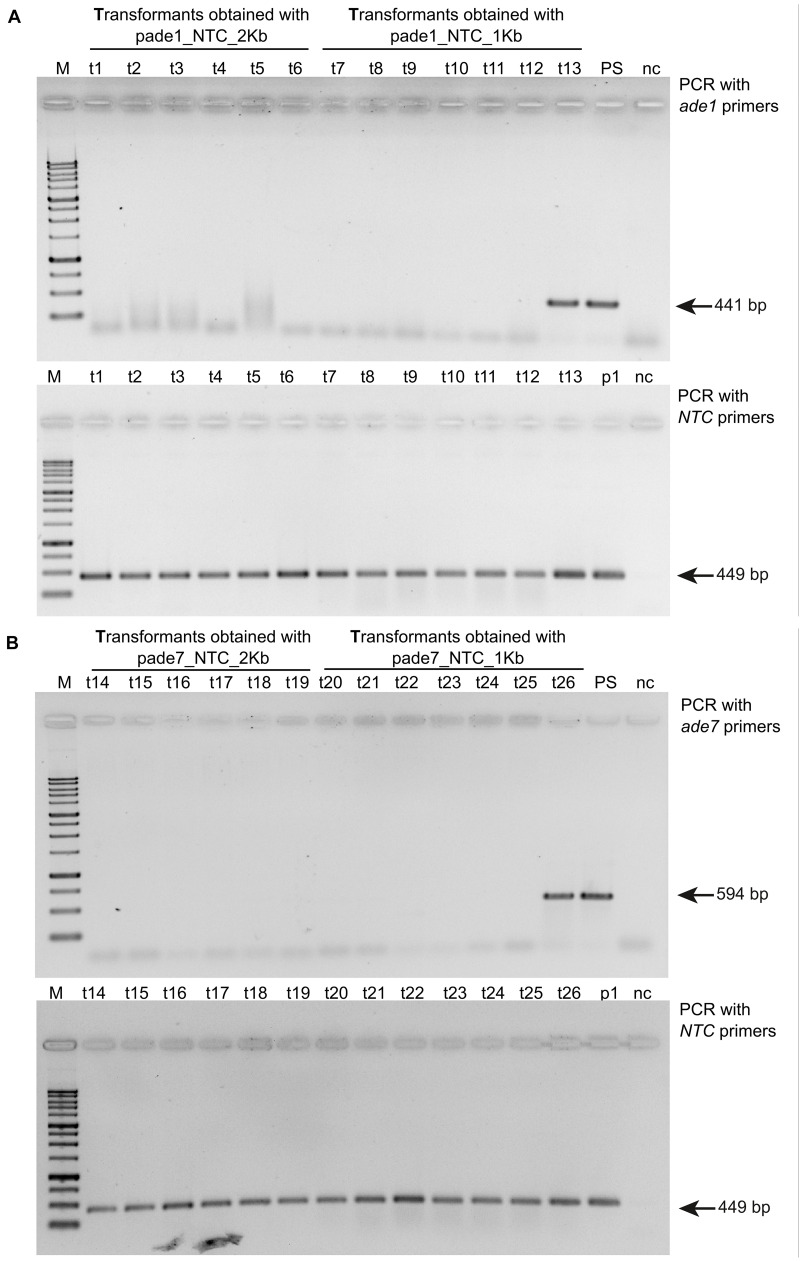
Gel images showing PCR confirmation of *ade1* or *ade7* deletion in *Ophiostoma novo-ulmi* transformants. **(A)** Total genomic DNA samples from twelve pink colony transformants and one white colony transformant (t13) were amplified with a*de1* specific primer pair ade1F-809 and ade1R-1221 (top panel) and *nat1* marker primers ntcF_562 and ntc_975_R (bottom panel). Lanes: M, 1 kb plus (FroggaBio); t1 through t6 and t7 through 13, 174_68Δ*mus52* transformants obtained using pade1_NCT_2kb and pade1_NTC_1kb, respectively; PS, parental strain 174_68Δ*mus52*; p1, pA-NTC-OSCAR; nc, negative H_2_O control. **(B)** Total genomic DNA samples from twelve pink colony transformants and one white colony transformant (t26) were amplified with a*de7* specific primer pair ade7F-368 and ade7R-809 (top panel) and *nat1* marker primers ntcF_562 and ntc_975_R (bottom panel). Lanes: M, 1kb plus (FroggaBio); t14 through t19 and t20 through 26, 174_68Δ*mus52* transformants obtained using pade7_NTC_2kb and pade1_NTC_1kb, respectively; PS, parental strain 174_68Δ*mus52*; p1, pA-NTC-OSCAR; nc, negative H_2_O control.

Six pink transformants for each deletion construct, which harbored the *nat1* cassette, were randomly chosen to be analyzed for successful gene deletion via PCR using primer pairs targeting either *ade1* or *ade7* (Supplementary Table 1). We confirmed deletion of *ade1* or *ade7* by HR in every pink colony examined (Figure 3). Additionally, the virulence of mutants ade1-6 *(*Δ*mus52*Δ*ade1*) and ade7-13 *(*Δ*mus52*Δ*ade7*) toward American elm saplings was tested. By 3 weeks post-inoculation, both progenitor strains (WT H327 and 174_68Δ*mus52*) had caused high levels of defoliation, whereas mutants ade1-6 (Δ*mus52*Δ*ade1*) and ade7-13 (Δ*mus52*Δ*ade7*) were impaired in their ability to induce DED (Table 1).

**TABLE 1:** Disease severity of *Ulmus americana* saplings inoculated with *Ophiostoma novo-ulmi* H327 wild-type, NHEJ-defective mutant strain 174_68Δ*mus52*, and two adenine auxotrophs (ade1-6 and ade7-13) derived from it by targeted deletion of genes *ade1* and *ade7*.

	**Disease severity**		
		**95% Confidence interval**	
**Treatment**	Percent*	**Lower limit**	**Upper limit**
Water	10.3 ^*a*^	7.8	12.8
*O. novo-ulmi* H327 (WT)	99.0 ^*b*^	96.5	100.0
174_68Δ*mus 52* (Δ*mus52*)	99.7 ^*b*^	97.2	100.0
ade1-6 (Δ*mus52*Δ*ade1*)	4.0 ^*a*^	1.0	7.0
ade7-13 (Δ*mus52*Δ*ade*7)	3.0 ^*a*^	0.2	6,2

*Median of percent disease severity (defoliation) at 21 days post inoculation. Different letters indicate statistically significant differences (*P* < 0.05) according to a Bonferroni test.

### Validation of the Efficiency of the Protocol Based on Using an NHEJ-Impaired Mutant of *Ophiostoma novo-ulmi* and HSVtk-Counterselection

Colonies of *O. novo-ulmi* strain 174_68Δ*mus52* transformed with OSCAR constructs containing 1 Kb of homologous arm length for genes encoding fungal binuclear TFs *bct2*, *ogf1* and o*pf2* (located on chromosomes 1 or 2) were grown on selection medium with nourseothricin and 5FU. PCR analysis of 17 transformants from each fungal transformation confirmed deletion of *bct2*, *ogf1*, and *opf2* in 13 (76%), 14 (82%), and 16 (94%) of the transformants tested, respectively (data not shown). These results confirmed that null mutants could be recovered at high frequency in *O. novo-ulmi* through an approach combining OSCAR-based counterselection with a Δ*mus52* mutant defective in NHEJ.

## Discussion

Recovery of null mutants is critical for assessing the functions and importance of genes in cellular processes. In fungi, gene knockout techniques by HR are a useful and popular functional genomics tool for generating null mutants (Qiao et al., 2019). However, HR frequencies in most fungi are extremely low, typically less than 5%, which has strongly limited gene function studies in these organisms (Scheffer et al., 2005; de Boer et al., 2010; Kück and Hoff, 2010). Several tools and strategies have been developed for genetic manipulation and applied in various fungi to either improve HR frequencies or simplify efforts required for production and screening of null mutants. In spite of advances in genetic transformation, a useful methodology for genetic manipulation is still lacking for many important phytopathogenic fungi, thereby restricting the validation of hypotheses generated by NGS-based approaches. During the last decade, genomic and transcriptomic studies have provided valuable data on cellular processes associated with development and virulence in the DED pathogens (Khoshraftar et al., 2013; Comeau et al., 2015; Nigg et al., 2015; Sbaraini et al., 2017). Nevertheless, molecular mechanisms involved in vegetative and parasitic growth of *Ophiostoma* species remain unexplored for lack of an efficient method of gene knockout (Bernier, 2016). In order to fully exploit the new knowledge obtained from NGS approaches and gain insight into molecular aspects underlying infection process and symptom induction by DED pathogens, we developed a high-efficiency gene deletion methodology by HR for *O. novo-ulmi*.

Efficiency of HR is mainly determined by two main DNA double-strand break repair pathways, namely HR and NHEJ (Krappmann, 2007). However, other parameters such as the genomic position of the target gene, homologous sequence length and transformation method can also influence HR efficiency (Michielse et al., 2005). Despite enhancement of HR frequency, ectopic integration can be frequent and therefore hinder greatly the selection of null mutants. This problem was solved successfully in several fungal species by using counterselection systems which greatly reduced the number of ectopic transformants (Khang et al., 2005; Wang et al., 2016; Twaruschek et al., 2018). For example, the latest version of the OSCAR method combines the rapid production of deletion constructs and the counterselection marker *HSVtk* compatible with ATMT (Paz et al., 2011; Sarmiento-Villamil et al., 2020). We thus took advantage of the high-throughput approach to gene deletion of the most recent OSCAR version to determine the effects of homologous arm length, impaired activity of NHEJ pathway and counterselection by *HSVtk* and 5FU on the generation of null mutants of *O. novo-ulmi* by HR. To establish an easily scorable reporting system for positive HR, we deleted *ade1* and *ade7*, critical genes in the purine pathway, which encode phosphoribosylamidoimidazole carboxylase and phosphoribosylaminoimidazole-succinocarboxamide synthase, respectively. *Ophiostoma novo-ulmi* mutants lacking either *ade1* or *ade7* produce colonies that turn pink over time as a result of the polymerization of aminoimidazole ribotide (an intermediate of adenine biosynthesis) (Bernier and Hubbes, 1990).

In *O. novo-ulmi*, targeted gene deletion has previously been achieved with an HR efficiency of less than 2% either by protoplast-mediated transformation (PMT) with recombinant plasmids in the presence of polyethylene glycol (Bowden et al., 1996; Temple et al., 2009) or treating whole cells in the yeast form with a deletion cassette in the presence of lithium acetate (Naruzawa, 2015). In several species of filamentous fungi, increased frequencies of HR were obtained by using ATMT (Michielse et al., 2005; Qiao et al., 2019). However, deletion of *ade1* in WT *O. novo-ulmi* by ATMT did not show a rise in HR frequency. A similar result had been reported earlier by Gardiner and Howlett (2004) who did not observe a dramatic difference in gene-targeting efficiency using ATMT compared to *PMT* in *Leptosphaeria maculans*.

An important feature of our approach was the use of *HSVtk* as a counterselection marker, which proved to be successful in enriching for gene deletion mutants in fungi such as *Magnaporthe oryzae*, *F. oxysporum*, *V. dahliae*, and *F. graminearum* (Gardiner and Howlett, 2004; Khang et al., 2005; Wang et al., 2016; Twaruschek et al., 2018). In spite of a high stability (over 82%) of *HSVtk* at the right border of T-DNA determined by Khang et al. (2005) and its high efficiency in counterselecting ectopic transformants in many Ascomycota species, *O. novo-ulmi* transformants were able to grow on PDA with hygromycin and 5FU and did not exhibit deletion of the target gene. These transformants mainly resulted from ectopic integrations of the truncated T-DNA, leading to the loss of *HSVtk* activity. Right-left border T-DNA truncations have been shown to occur in fungi recalcitrant to gene deletion by HR. Interestingly, genome sequence analyses in random mutants from *L. maculans* obtained by ATMT showed all T-DNA borders were truncated to different extents, including loss of the whole right border (Chambers et al., 2014). Martínez-Cruz et al. (2017) found similar results after they examined border sequences of the T-DNA regions of *Podosphaera xanthii* mutants. Additionally, there is evidence that truncation of T-DNA border sequences is common in ATMT. For example, the low rate of success in identifying insertions in *Trichoderma reesei* and the ascomycete plant pathogens *Colletotrichum higginsianum* and *M. oryzae* (Choi et al., 2007; Zhong et al., 2007; Huser et al., 2009) has been attributed to this phenomenon. Truncation of T-DNA could therefore explain why counterselection by *HSVtk* failed in WT *O. novo-ulmi*.

The frequency of HR varies considerably among fungal species, ranging from 1% in *Blastomyces dermatitidis* to almost 100% in *S. cerevisiae* (Brandhorst et al., 1999; Gauthier et al., 2010). However, as mentioned previously, low HR frequency in fungi can be improved considerably by blocking NHEJ activity, which is typically achieved by replacing/inactivating one of the genes in this pathway, such as *mus51*/*ku70*, *mus52*/*ku80*, or *mus53*/*lig4* (Maier et al., 2005; Meyer et al., 2007; Huang et al., 2017). Use of NHEJ-defective strains has been shown to promote very efficient (up to 100%) targeted integration at the homologous locus by HR (Ninomiya et al., 2004; Krappmann et al., 2006; El-Khoury et al., 2008; Li et al., 2010). However, only 2 of 84 transformants derived from a NHEJ-defective strain of *O. novo-ulmi* H327 lacking gene *mus52* showed correct gene replacement of *ppo1*. This low rate of HR (2.4%) was associated with the short length (approximately) 500 bp of flanking regions used to delete *ppo1* (Naruzawa, 2015). By increasing the length of flanking regions to 1 Kb and 2 Kb when transforming *O. novo-ulmi* strain 174_68Δ*mus52*, we were able to increase the frequency of targeted gene replacement to 30 and 47%, respectively. In *N. crassa* lacking *mus52*, HR frequency increased to over 90% when homologous arm length was 500 bp (compared to 9% in WT) and reached 100% when homologous arm length was 1 Kb (compared to 21% in WT) (Ninomiya et al., 2004). In a *ku80* gene disruption mutant of the human pathogen *C. neoformans*, HR frequency increased to 3% when 0.1 Kb homologous flanks were used (compared to 0% in WT) and to 64% with 1 Kb homologous flanks (compared to 9% in WT) (Li et al., 2010). In mutants of *Aspergillus chevalieri*, *Aspergillus niger*, and *N. crassa* lacking *ku70* or *lig4*, the efficacy of HR was also shown to be influenced by the length of homologous sequences in the deletion construct (Ninomiya et al., 2004; Ishibashi et al., 2006; Meyer et al., 2007; Huang et al., 2017). Our results confirmed that NHEJ plays a major role in HR and that efficacy depends on the length of homologous arms that flank the gene targeted for deletion. However, in spite of the significant increase in HR frequency resulting from *mus52* deletion in *O. novo-ulmi*, a high rate of random integration of the T-DNA still occurred in strain 174_68Δ*mus52*, thereby suggesting the preferential use of the NHEJ over the HR pathway in *O. novo-ulmi.*

According to the major model of foreign DNA integration in the host genome, dsDNA is recognized and recruited by the double-strand break repair machinery, mostly by NHEJ (Krappmann, 2007; Gelvin, 2017). This may be due to the fact that the NHEJ pathway is active in all phases of the cell cycle and represents the simplest and fastest mechanism for foreign dsDNA integration (Krappmann, 2007; Lieber, 2010; Chang et al., 2017). Ku70 and Ku80 proteins initiate NHEJ, capping the free dsDNA ends, and physically block the HR pathway (Langerak et al., 2011; Shao et al., 2012). During formation of foreign DNA-heterodimer Ku70/Ku80 complex and integration event, deletions of foreign DNA could take place (Gelvin, 2017). Hence, we suspected that counterselection by *HSVtk* could work in *O. novo-ulmi* lacking *mus52*. A comparison of 174_68Δ*mus52* ectopic transformants recovered from selective medium, either supplemented with 5FU or without, showed a drastic reduction (up to 94%) of transformants in the presence of 5FU. These results suggest that Ku80 intervenes in the deletion of foreign DNA segments during the genomic integration process. Interestingly, coupling of ATMT with *HSVtk*-based counterselection worked efficiently for enrichment of gene replacement mutants and prevention of ectopic transformants in strain 174_68Δ*mus52*, even with 1 Kb of flanking sequence. Additionally, successful targeted deletion of five genes, located on four different chromosomes, suggests that the ATMT-OSCAR-*HSVtk* strategy is not restricted to certain loci.

Colonies formed by Δ*ade1* or Δ*ade7* mutants exhibited the pink phenotype observed previously in some of the *O. novo-ulmi* adenine-requiring strains obtained by chemical mutagenesis (Bernier and Hubbes, 1990). In addition, inoculation of strains ade1-6 and ade7-13 to young American elm saplings showed that the mutants had lower virulence. This phenotype was not due to impairment of the NHEJ pathway in the mutants since the virulence of progenitor strain 174_68Δ*mus52* was high and comparable to that of WT strain H327. Reduction of virulence resulting from auxotrophy for adenine has previously been reported in several plant pathogenic fungi including *F. oxysporum* f. sp. *melonis* (Denisov et al., 2005), *Gibberella zeae* (Kim et al., 2007), and *C. higginsianum* (Korn et al., 2015). The virulence of knockout mutants for genes *bct2*, *ogf1* and *opf2* will be evaluated when elm saplings are available for pathogenicity tests.

Until now, functional genomic analysis of *O. novo-ulmi* was limited by the absence of an efficient gene deletion tool. This is no longer the case, as we were able to recover from 64 to 94% of targeted deletion mutants among transformants derived from NHEJ-defective strain 174_68Δ*mus52* subjected to a dual-selection system in OSCAR. Therefore, systematic gene function studies can now be envisioned for elucidating the molecular bases of pathogenicity and virulence in the DED fungi.

## Data Availability Statement

The original contributions presented in the study are included in the article/Supplementary Material, further inquiries can be directed to the corresponding authors.

## Author Contributions

JLS-V, TCO, and LB designed the study. ESN constructed the *Ophiostoma novo-ulmi* strain 174_68Δ*mus52* used in the improved OSCAR-based protocol. JLS-V and TCO constructed the gene delete OSCAR plasmids and performed transformation experiments, DNA extractions molecular analyses, and phenotyping experiments. LB carried out the inoculations on elm saplings and supervised the research. JLS-V wrote the manuscript with input from all authors. All authors contributed to the article and approved the submitted version.

## Conflict of Interest

The authors declare that the research was conducted in the absence of any commercial or financial relationships that could be construed as a potential conflict of interest.
