# Supplementary material for: An Efficient Strategy for Obtaining Mutants by Targeted Gene Deletion in Ophiostoma novo-ulmi
Source: Front Microbiol. 2021 Jul 14;12:699783. doi: 10.3389/fmicb.2021.699783 (PMC8317267; doi:10.3389/fmicb.2021.699783)
Supplement: Supplementary file 1 [file Table_1.docx]

Supplementary Material

| **Supplementary table S1.** Primers used to amplify specific gene fragments | | |  |
| --- | --- | --- | --- |
|  |  |  | |
| Primer | **5´-3´Primer sequence** |  | |
| Ade1F_-3072_attB2r | ***GGGGACAGCTTTCTTGTACAAAGTGGAA***ACGAGACGATTCTGCGTAGC | Forward primer used for amplification of 3 Kb of the downstream region of *ade1* with site-specific recombination (attB2r) to produce the deletion construct | |
| Ade1F_-2088_attB2r | ***GGGGACAGCTTTCTTGTACAAAGTGGAA***GCCTGGACCAGTATGAGATG | Forward primer used for amplification of 2 Kb of the downstream region of *ade1* with site-specific recombination (attB2r) to produce the deletion construct | |
| Ade1F_-1120_attB2r | ***GGGGACAGCTTTCTTGTACAAAGTGGAA***AACAAACGATCTGAGTGGCC | Forward primer used for amplification of 1 Kb of the downstream region of *ade1* with site-specific recombination (attB2r) to produce the deletion construct | |
| Ade1R_-1_attB1r | ***GGGGACTGCTTTTTTGTACAAACTTGT***TGTTAAGAGATTTTGGGTATATG | Reverse primer used for amplification of the downstream region of *ade1* with site-specific recombination (attB1r) to produce the deletion construct | |
| Ade1F_1870_attB4 | ***GGGGACAACTTTGTATAGAAAAGTTGTT***ATACTCAAAGTGTTACATAGC | Forward primer used for amplification of the upstream region of *ade1* with site-specific recombination (attB4) to produce the deletion construct | |
| Ade1R_2949_attB3 | ***GGGGACAACTTTGTATAATAAAGTTGT***CGGTAAGGAGGTTGTGTACG | Reverse primer used for amplification of 1 Kb of the upstream region of *ade1* with site-specific recombination (attB3) to produce the deletion construct | |
| Ade1R_3854_attB3 | ***GGGGACAACTTTGTATAATAAAGTTGT***CTCTCCATCTTCCGCCTTTG | Reverse primer used for amplification of 2 Kb of the upstream region of *ade1* with site-specific recombination (attB3) to produce the deletion construct | |
| Ade1R_4936_attB3 | ***GGGGACAACTTTGTATAATAAAGTTGT***TTGTCGCGAGCCATAATGTTG | Reverse primer used for amplification of 3 Kb of the upstream region of *ade1* with site-specific recombination (attB3) to produce the deletion construct | |
|  |  |  | |
| Ade1F_203 | ATGTCCTGACCGTCGAGATTG | Primers used for amplification of *∆ade1* fragment | |
| Ade1R_836 | GTCTTGGTGACAAACAGCTCG |  |  |
| Ade7F_-2975_attB2r | ***GGGGACAGCTTTCTTGTACAAAGTGGAA***CACAAAGAAGAGGCCGTTG | Forward primer used for amplification of 3 Kb of the downstream region of *ade7* with site-specific recombination (attB2r) to produce the deletion construct | |
| Ade7F_-1932_attB2r | ***GGGGACAGCTTTCTTGTACAAAGTGGAA***GATACATGCAGAACCTGGGG | Forward primer used for amplification of 2 Kb of the downstream region of *ade7* with site-specific recombination (attB2r) to produce the deletion construct | |
| Ade7F_-1027_attB2r | ***GGGGACAGCTTTCTTGTACAAAGTGGAA***GATGCATGTGCGGAGAAGAG | Forward primer used for amplification of 1 Kb of the downstream region of *ade7* with site-specific recombination (attB2r) to produce the deletion construct | |
| Ade7R_-1_attB1r | ***GGGGACTGCTTTTTTGTACAAACTTGT***GGTGACGTAATGGTTAGATAGG | Reverse primer used for amplification of the downstream region of *ade7* with site-specific recombination (attB1r) to produce the deletion construct | |
| Ade7F_967_ attB4 | ***GGGGACAACTTTGTATAGAAAAGTTGTT***ATAGGTAGAGCGCAGATAG | Forward primer used for amplification of the upstream region of *ade7* with site-specific recombination (attB4) to produce the deletion construct | |
| Ade7R_ 4048_attB3 | ***GGGGACAACTTTGTATAATAAAGTTGT***GCTCAGGCATACACACCAC | Reverse primer used for amplification of 1 Kb of the upstream region of *ade7* with site-specific recombination (attB3) to produce the deletion construct | |
| Ade7R_ 3050_attB3 | ***GGGGACAACTTTGTATAATAAAGTTGT***AGCCTAATCGCTGTCACGG | Reverse primer used for amplification of 2 Kb of the upstream region of *ade7* with site-specific recombination (attB3) to produce the deletion construct | |
| Ade7R_ 2077_attB3 | ***GGGGACAACTTTGTATAATAAAGTTGT***GATTGTGAGCATTGGCACTC | Reverse primer used for amplification of 3 Kb of the upstream region of *ade7* with site-specific recombination (attB3) to produce the deletion construct | |
| Ade7F_55 | AAGGTTCGCGAGATGTTTGTC | Primers used for amplification of *∆ade7* fragment | |
| Ade7R_628 | CAGTGTAGACAGCAAGAGCC |  |  |
|  |  |  | |
| Bct2F_-1050_attB2r | ***GGGGACAGCTTTCTTGTACAAAGTGGAA***TTCCCAAACGGGCTCTATTC | Primers used for amplification of the downstream region of *bct2* with site-specific recombination to produce the deletion construct | |
| Bct2R_-1_attB1r | ***GGGGACTGCTTTTTTGTACAAACTTGT***AATGAGGGAAAATGGCTATG |  |  |
| Bct2F_1890_ attB4 | ***GGGGACAACTTTGTATAGAAAAGTTGTT***TTTTGTTATTCTCTCATTGCC | Primer used for amplification of the upstream region of *bct2* with site-specific recombination to produce the deletion construct | |
| Bct2R_2903_attB3 | ***GGGGACAACTTTGTATAATAAAGTTGT***GGGTCTGTCGCACATTATC |  |  |
| Bct2F_539 | TCCACCTCCACATTCAAGACG | Primers used for amplification of *∆bct2* fragment | |
| Bct2R_1111 | AAACCCATCTTTTGCTGACGC |  |  |
| Ogf1F_-1120_attB2r | ***GGGGACAGCTTTCTTGTACAAAGTGGAA***CTACTGCCTCTTGCCTCTTCTC | Primers used for amplification of the downstream region of *ogf1* with site-specific recombination to produce the deletion construct | |
| Ogf1R_-4_attB1r | ***GGGGACTGCTTTTTTGTACAAACTTGT***GTAGGTGTTTGTAGATGTGTAG |  |  |
| Ogf1F_2850_attB4 | ***GGGGACAACTTTGTATAGAAAAGTTGTT***AGTTCTTCTGTGAAGGGTAC | Primers used for amplification of the upstream region of *ofg1* with site-specific recombination to produce the deletion construct | |
| Ogf1R_3920_attB3 | ***GGGGACAACTTTGTATAATAAAGTTGT***TCCTTCCTCGCCACAATCTTC |  |  |
| Ogf1F_506 | CTGCTATGCGCGTTTCTTAGG | Primers used for amplification of *∆ogf1* fragment | |
| Ogf1R_1133 | GGATGGTCTTGGAGATGACCC |  |  |
| Opf2F_-1012_attB2r | ***GGGGACAGCTTTCTTGTACAAAGTGGAA***TCGACAATGCTCATCGTCTTAC | Primers used for amplification of the downstream region of *opf2* with site-specific recombination to produce the deletion construct | |
| Opf2R_-1_attB1r | ***GGGGACTGCTTTTTTGTACAAACTTGT***GGTGGGAGTTCAGAGGTTGAAG |  |  |
| Opf2F_2446_attB4 | ***GGGGACAACTTTGTATAGAAAAGTTGTT***TTGCTTATGCCAGGGACGGACC | Primers used for amplification of the upstream region of *opf2* with site-specific recombination to produce the deletion construct | |
| Opf2R_3468_attB3 | ***GGGGACAACTTTGTATAATAAAGTTGT***TGCTCCTCACATTCCTTACCG |  |  |
| Opf2R_254 | TTGCTCCAACTTCGGGTCTAC | Primers used for amplification of *∆opf2* fragment | |
| Opf2R_873 | GTTCGCGTATGGGTCATTTGG |  |  |
|  |  |  | |
|  |  |  | |
| NatF_562 | TGTCGTCAAGAGTGGTCATGG | Primers used for amplification of nourseothricin resistance cassette fragment | |
| NatR_1110 | GGTGTATGAAACCGGAAAGGC |  |  |
| HygF_Oscar | AAAGCCTGAACTCACCGCGACG | Primers used for amplification of hygromycin resistance cassette fragment | |
| HygR_Oscar | AGCGCGTCTGCTGCTCCATAC |  |  |
|  |  |  | |
